# Establishment of a risk assessment tool for pregnancy-associated venous thromboembolism and its clinical application: protocol for a prospective observational study in Beijing

**DOI:** 10.1186/s12884-019-2448-7

**Published:** 2019-08-13

**Authors:** Yi Chen, Yan Dai, Jing Song, Ling Wei, Ying Ma, Ning Tian, Qian Wang, Qian Zhang, Yue Zhang, Xiao Lan Wang, Jun Zhang, Rong Liu

**Affiliations:** Beijing Obstetrics and Gynecology Hospital, Capital Medical University, 251# Yao Jia Yuan Road, Chao Yang District, Beijing, 100026 China

**Keywords:** Venous thromboembolism, Pregnancy-associated, Pregnancy and puerperal

## Abstract

**Background:**

The risk of venous thromboembolism (VTE) during pregnancy and puerperal periods is significantly higher than during the non-pregnant period and is one of the major causes of maternal mortality. Developed countries have promulgated guidelines for risk assessment and prevention of maternal VTE, and standardized management has led to a significant reduction in maternal mortality. However, there is a paucity of relevant research related to pregnancy and puerperal VTE in China.

**Methods/design:**

We will perform a prospective cohort study and recruit 13,000 pregnant women from 2018 to 2020 in Beijing, China. VTE risk assessment will be conducted using the Royal College of Obstetricians and Gynaecologists (RCOG) pregnancy and puerperal VTE risk-assessment-scoring tool during early and late pregnancy, as well as during the puerperal period. Venous ultrasonography of lower extremities, routine blood tests, and coagulation parameters will be examined. These VTE risk assessments will be performed again if patients have VTE-related symptoms during their pregnancies, or if any of the following occur: (1) patients are hospitalized over 7 days due to any pregnancy complications; (2) patients are placed under strict bed rest for ≥ 3 days to prevent miscarriage. For patients with a confirmed diagnosis of VTE, treatment and follow-up plans will be decided jointly by the obstetricians, vascular surgeons, and pulmonologists. All patients in the study will be followed up by dedicated healthcare providers for up to 42 days postpartum. Statistical analyses will be performed to test the feasibility of the RCOG scoring tool for the Chinese population. The RCOG scoring tool will then be revised based upon the characteristics of the Chinese population, and the revised assessment scoring tool will then be tested in the cohort to evaluate its efficacy. Finally, a pregnancy and puerperal VTE risk-assessment tool will be proposed based on our study results.

**Discussion:**

This study will establish a preliminary VTE risk-assessment tool that is applicable to pregnant and puerperal women in China and provide guidelines for further thrombophylactic interventions. Furthermore, we wish to draw increased attention to pregnancy-associated VTE to reduce VTE-related mortality.

**Trial registration:**

Chi CTR1800015848 (04/24/2018).

## Background

Pregnant and puerperal women face a higher risk for venous thromboembolism (VTE) than that of non-pregnant women due to their specialized physiologic changes, including hypercoagulation, venous stasis, and vascular endothelial injury. Strikingly, VTE is one of the major causes of maternal mortality [1–3]. VTE primarily includes deep-vein thrombosis (DVT) and pulmonary embolism (PE) and, if not diagnosed and treated promptly, 15–24% of patients with DVT will progress to PE, which is a significant factor in sudden death. Studies from developed countries have reported that the relative risk of VTE during the pregnant and puerperal periods is nearly 20 times higher than during the non-pregnant period [4]. which can impose fatal effects on pregnant women. In various countries (including the United States and the United Kingdom), the percent of all maternal deaths that are attributed to VTE once reached 15–31.1% [5, 6]. Although VTE is a fatal disease, it is preventable [7–10]. In western countries, guidelines and a consensus have been established based on evidence-based medicine directing the VTE risk assessment for all pregnant women. Low-molecular-weight heparin or other anticoagulants, as well as mechanical prophylactic devices, have been applied to high-risk pregnant women to reduce the incidence of VTE. The World Health Organization reported that maternal deaths due to thromboembolic disease declined from 14.8% in 2006 to 3.2% in 2014 [11, 12]. Currently, the Royal College of Obstetricians and Gynaecologists (RCOG), the American College of Obstetricians and Gynecologists (ACOG), and the Working Group in Women’s Health of the Society of Thrombosis and Haemostasis (GTH) all recommend that every woman of child-bearing age be assessed for VTE risk during preconception, pregnancy, and puerperal periods. Appropriate prophylactic treatments should then be administered to women with a high risk for thrombosis [13–15].

The study of pregnant and puerperal VTE in China lags behind that of more developed countries, and there is limited empirical evidence and clinical experience reported. There are also no standardized clinical guidelines, consensuses, or administrative systems in place to assess, screen, and prevent VTE. With the adjustment in China’s family-planning strategy and release of the second-child policy, the risk factors for VTE—including advanced maternal age, use of assisted reproductive technology, multiple pregnancy, obstetric complications, and cesarean sections—have increased. However, insufficient attention is currently being paid to pregnancy-associated VTE. A study performed in a Chinese population showed that nearly half of patients with DVT manifested no clinical symptoms [16] and that, during pregnancy, lower-extremity edema further masked clinical presentations of DVT. This may lead to delayed diagnosis and treatment of DVT, which may then further develop into PE and ultimately cause death in pregnant women.

At present, the incidence of pregnancy-associated VTE in China is unknown, and the risk factors associated with this condition are unclear. Due to the majority of Han ethnic background of the Chinese population, genetic factors play limited roles in VTE in China. Common risk factors for VTE—such as history of multiple deliveries and cigarette smoking—are also less frequently observed in China. However, some common practices such as traditional puerperal “confinement,” strict bed rest during pregnancy to prevent miscarriage, and cesarean section occur more often in China and are risk factors for VTE. These factors suggest that the risk-assessment tool used in western countries may not be applicable to China. In addition, our medical technology, economic development, and traditional habits are different from those in western countries. It is therefore unknown whether we can apply to the Chinese population the risk-assessment tool developed by western countries. It is also unclear whether we can give thrombophylactic treatments to high-risk Chinese patients based on standards developed in western countries.

Therefore, there is an urgent need to understand the risk factors for pregnancy-associated VTE in China, and to establish a preliminary VTE risk-assessment tool for pregnant Chinese women. Such a tool would provide guidelines for clinicians to evaluate VTE risk and initiate appropriate thrombophylactic interventions. Use of the tool may decrease the incidence of VTE, prevent serious complications (or even death) in pregnant women, and improve overall pregnancy outcomes.

## Methods/design

### Ethics statement

The present study was approved by the ethics committee of Beijing Maternity Hospital, which is affiliated with Capital Medical University in Beijing, China. Informed consent will be obtained from the patients or their families.

### Aims

#### Primary aims

This study aims to meet two primary objectives as follows. Our first objective is to establish a prospective study cohort to assess VTE risks by the VTE risk-assessment tool from the 2015 RCOG document entitled “Reducing the Risk of Venous Thromboembolism during Pregnancy and the Puerperium.” We will estimate the percent of their study population who would be eligible for VTE prophylaxis under the UK RCOG guidelines based on their risk-factor profile and test whether the tool, validated in western countries, can be applied to the Chinese population. We will perform compression-duplex ultrasound of the bilateral lower extremities on every patient, as well as quantify the prevalence of asymptomatic VTE and how many of these events become clinically apparent. Our second objective is to propose a newly revised risk-assessment tool based upon the Chinese population. The cohort in the current study will thus be used to evaluate the clinical applicability of this newly revised risk-assessment tool.

#### Secondary aims

Based on the study results, and with consideration of expert opinions, a new pregnancy and puerperal VTE risk-assessment tool for the Chinese population will be developed, so as to provide clinical guidelines for thrombophylactic treatment.

### Study design

This will be a prospective observational cohort study aimed at investigating VTE during pregnancy. VTE risk assessment will be performed during early and late pregnancy, as well as in the puerperal period (based upon the RCOG pregnancy and puerperal VTE risk-assessment tool). This risk-assessment form is given in Table 1. Compression-duplex ultrasound, routine blood tests, and coagulation-function tests will be undertaken in our hospital. If the diagnosis of VTE is confirmed, treatment and a follow-up plan will be decided after consultation with the vascular surgeons. For those patients who develop VTE-related symptoms, are hospitalized over 7 days due to pregnancy complications, or are restricted to bed rest ≥ 3 days to prevent miscarriage or for other reasons, we will repeat the VTE risk assessment, compression-duplex ultrasound, routine blood tests, and coagulation-function tests. As mentioned above, once the diagnosis of VTE is confirmed, treatment and a follow-up plan will be decided after consultation with the vascular surgeons. We will execute statistical analyses based on the clinical data to verify the feasibility of using the RCOG scoring tool in China. Risk factors in the RCOG tool (but not associated with VTE in the Chinese population) will be identified, as well as novel risk factors for the Chinese population. Then, a newly revised VTE risk-assessment tool will be proposed by modifying the RCOG tool, and this new tool will be tested in the cohort patients to evaluate its efficacy. Finally, based on the study results and consideration of expert opinions, points will be assigned to every risk factor in the revised assessment tool. We will thereby develop a pregnancy-associated VTE risk-assessment tool to be used in China.

**Table 1:** Risk-assessment form for venous thromboembolism (VTE)

First trimester risk factors assessment	Score Tick
Previous VTE (except a single event related to major surgery)	4
Previous VTE provoked by major surgery	3
Known high-risk thrombophilia	3
Medical comorbidities e.g. cancer, heart failure; active systemic lupus erythematosus, inflammatory polyarthropathy or inflammatory bowel disease; nephrotic syndrome; type I diabetes mellitus with nephropathy; sickle cell disease; current intravenous drug user	3
Family history of unprovoked or estrogen-related VTE or postpartum thromboin first-degree relative	1
Age (> 35 years)	1
Obesity	1 or 2 a
Parity ≥3	1
Smoker	1
Gross varicose veins	1
ART/IVF	1
Multiple pregnancy	1
Hyperemesis	1
OHSS	1
Immobility (strict bed rest ≥3 days), dehydration	1
Current systemic infection	1
Total	
Second & Third trimester risk factors assessment	
Hospital admission (> 7 days)	1
Any surgical procedure e.g. appendicectomy	1
Current pre-eclampsia	1
Total	
Postnatal assessment risk factors assessment	
Caesarean section in labor	2
Elective caesarean section	1
Mid-cavity or rotational operative delivery	1
Prolonged labor (> 24 h)	1
PPH (> 1 l or transfusion)	1
Preterm birth < 37 + 0 weeks in current pregnancy	1
Stillbirth in current pregnancy	1
Total	

a: BMI ≥ 30 = 1; BMI ≥ 40 = 2

### Study population

**Fig. 1:**
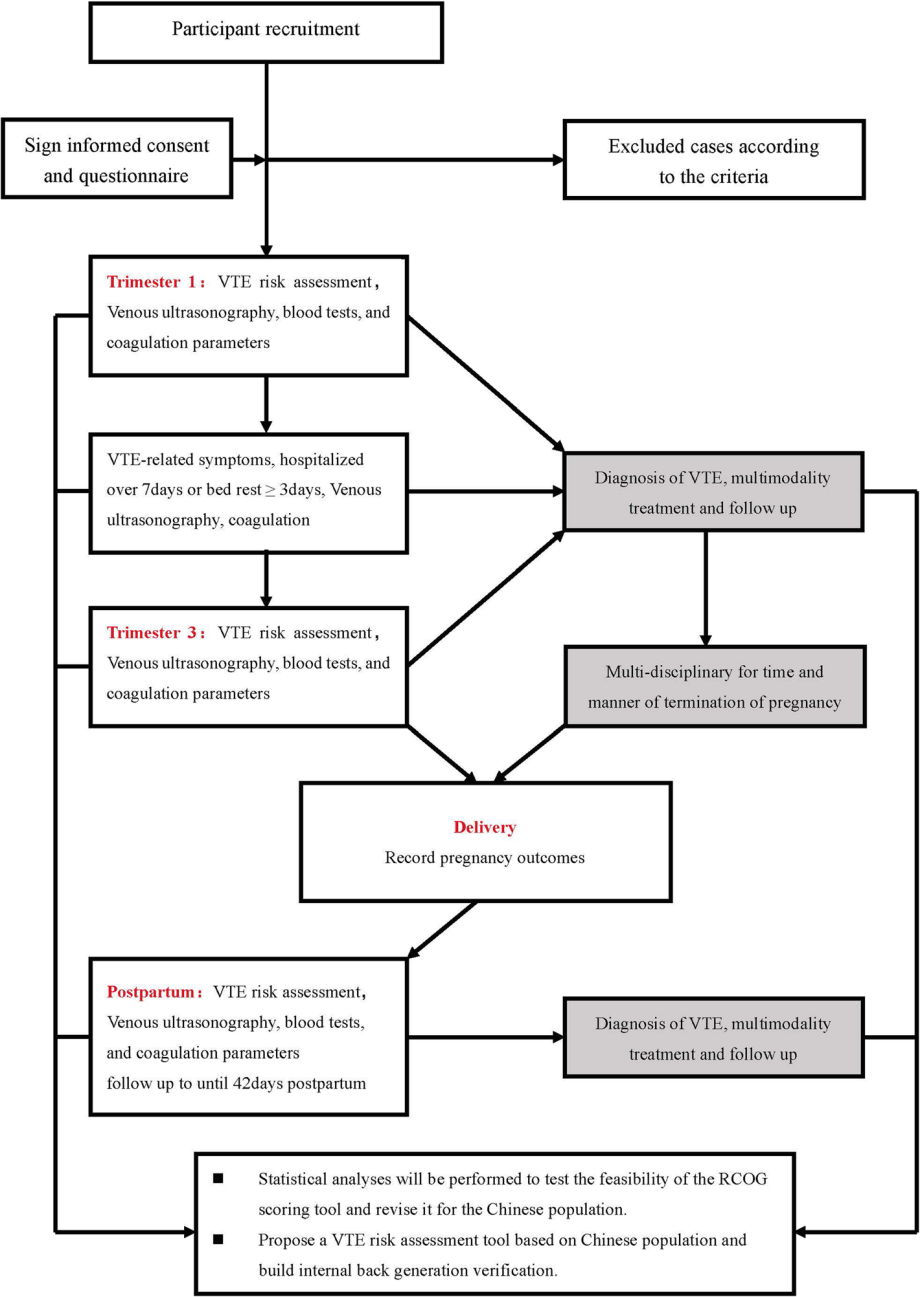
Flow Chart of the study. Establishment of a risk-assessment tool for pregnancy-associated venous thromboembolism and its clinical application

Pregnant patients who visit the Beijing Maternity Hospital affiliated with Capital Medical University between December 2018 and May 2020 will be invited to participate in this observational cohort study with a target number of 13,000 patients. Patient enrollment will be voluntary and involve detailed observations and evaluation. Pregnant women will be recruited when the ultrasound clarifies intrauterine pregnancy from 6 to 12 gestational weeks. Patients will sign the informed consent for regular prenatal and follow-up examinations until six-weeks postpartum. A flow chart of this trial is given in Fig. 1.

### Exclusion criteria

Exclusion criteria are as follows: (1) miscarriage before 12 weeks of gestational age; (2) severe heart, liver, or kidney dysfunction, as well as hematologic or immune system disorders; (3) patients with visual or mental disabilities.

### Diagnostic criteria for VTE

Lower-extremity DVT will be diagnosed if there is widening of the venous lumen, loss of compressibility, absence of blood-flow signals, or blood-filling defects under compression ultrasonography. DVT will also be diagnosed if there is no increase, a decrease, or a disappearance of blood-flow signals when compression is applied to the distal extremities.

PE will be diagnosed based on computed-tomography pulmonary angiography (CTPA), radionuclide ventilation/perfusion (V/Q) scanning, or magnetic-resonance pulmonary angiography.

## Data collection and management

### Sample-size calculation

The sample-size calculation was based on the diagnostic test for VTE, based on α = 0.05, β = 0.2, a minimal acceptable sensitivity of 55%, an expected sensitivity of 80%, a minimal acceptable specificity of 70%, and an expected specificity of 99%. The sample size was thus calculated to be 28 using PASS software. However, when considering a 10% loss to follow-up, 31 patients will be needed. If the incidence of VTE is 0.24%, a total of 13,000 pregnant women will be required for this cohort study.

### Collection of participant data

After informed consent is signed by the study participant, trained staff members will complete the study questionnaires, which include questions on age, height, weight, occupation, level of education, financial status, health situation, lifestyle, smoking/alcohol history, pregnancy history, past medical history, family history, method used to conceive, early pregnancy, hyperemesis, and administration of progesterone to prevent miscarriage. Pregnant women will enter the cohort during early pregnancy, and complete the follow-up examinations during the pregnant period and until 42 days postpartum. Medical records—including clinical presentations, laboratory tests, B-ultrasound images, clinical treatments, and prognosis—will be documented. We will establish a medical database to cover the entire pregnancy and neonatal periods from 13,000 mother–child pairs.

### Patient perspective measures

VTE-related genetic or acquired risk factors will be evaluated during early pregnancy (from 6 to 12 weeks of gestation), late pregnancy (after 34 weeks of pregnancy), and puerperal (3–7 days after birth) periods. The evaluation will be based on the RCOG pregnancy and puerperal VTE risk-assessment tool [13]. All pregnant women will receive compression-duplex ultrasound on the bilateral lower extremities, routine blood tests, and coagulation-function tests. If DVT is diagnosed, the treatment strategy—including low-molecular-weight heparin injection—will be decided after consultation with the vascular surgeons. Dedicated staff will continue to follow up with patients, and pregnant women without VTE will receive education on VTE prevention. For patients who develop VTE-related symptoms during pregnancy, are hospitalized over 7 days due to pregnancy complications, or are ordered to strict bed rest ≥ 3 days to prevent miscarriage or for other reasons, we will repeat the VTE risk assessment, compression-duplex ultrasound, routine blood tests, coagulation-function tests, and other necessary imaging studies. If the diagnosis of DVT is confirmed, the treatment strategy—including low-molecular-weight heparin injection—will be decided after consultation with the vascular surgeons. Designated staff will continue to follow up with patients, and if the diagnosis of PE is suspected, pulmonologists will be consulted. The treatment strategy and follow-up plan will be decided, and all other complications during the pregnancy will be recorded.

### Outcome measurements

All patients with a confirmed diagnosis of VTE will be followed up by dedicated staff for the types, doses, and durations of medications given. The time to pregnancy termination and mode of delivery, as well as the duration of anticoagulation therapy and follow-up time, will be decided based on the patient’s condition and a discussion among obstetricians, vascular surgeons, and pulmonologists.

After giving birth, all patients will have detailed records for their gestational weeks, mode of delivery, birth time, puerperal complications, requirement for bed rest ≥ three-days postpartum (or lack thereof), condition of the neonate, and outcomes of mothers and children. All pregnant women will be followed up until 42-days postpartum.

## Data analysis

We will use SPSS 22.0 software for statistical analyses. Descriptive data will be presented as the number of cases, means, standard deviation, and medians. The percent of patients in our study population who would be eligible for VTE prophylaxis under the UK RCOG guidelines based on their risk factor profile will be estimated. Multivariate logistic-regression analysis will be used to test the efficacy of the RCOG pregnancy and puerperal VTE risk-assessment tool in the current cohort and to provide the basis for thromboprophylaxis according to the risk factors of RCOG. Risk factors associated with VTE in the Chinese population will then be obtained to revise the RCOG scores and the revised risk-assessment tool will be tested by back-generation internal certification in the current cohort population. The sensitivity and specificity of the risk factors in the revised VTE risk-assessment tool, as well as the cut-off values, will be calculated. Diagnostic values based on the ROC curve will be constructed with MedCalc software. A *P* < 0.05 will be considered statistically significant. Based upon study results, and with the consideration of expert opinions, points will be given to each risk factor in the revised risk-assessment tool, and a pregnancy and puerperal VTE risk-assessment tool will ultimately be developed for China. Then, we will quantify the prevalence of asymptomatic VTE and how many of these events become clinically apparent, as well as analyze the reason for them.

## Quality control

First, a quality control team will be developed, with the principal investigator as the team leader. Second, prior to the start of the project, all research personnel will be trained to collect patient information, and dedicated staff will be assigned to collect and record data. Clinicians will check for logical errors and missing information and correct these within 24 h after completing the questionnaire. Data collection will use a paper version of the questionnaire, and data will also be later entered into a computerized database. Data quality will be evaluated at the same time. We will provide corresponding standards for quality control, follow-up plan, administrative management, and biologic-sample collection to ensure high overall quality and minimal biases in the current project. Third, once VTE is diagnosed, the research team personnel—including obstetricians, vascular surgeons, and pulmonologists—will discuss and create the individualized diagnosis and treatment plan, while maintaining the project principle in order to preserve the consistency of diagnosis and treatment throughout the entire project. Fourth, evaluation during the peripartum period will be conducted by attending physicians. Fifth, education will be provided to the pregnant women and their families to improve compliance and reduce the drop-out rate. Benefits of being enrolled in the study will include more comprehensive monitoring, evaluation, and follow-up to ensure safety of the pregnant women.

## Risk

There are certain risks for patients with confirmed VTE. Anticoagulation treatment—including selection of anticoagulants, doses, duration of treatment, follow-up plan, time to pregnancy termination, and models of delivery—will be decided after joint discussions among obstetricians, vascular surgeons, and pulmonologists. Particularly for those patients with serious DVT or PE, treatment will be individualized after multidisciplinary discussions. Patients and families will be notified of the diagnosis and treatment plans, and dedicated staff will be assigned to follow up with patients during the pregnancy and until 42 days after delivery to minimize the risk of severe complications.

## Patient and public involvement

No patients were involved in developing the plan for the design of the study or influencing the research questions. No patients were involved in the interpretation of study results or writing up of the manuscript. The study results will be disseminated to the study participants or the relevant patient community through social media and the pregnancy school in our hospital.

## Feasibility and clinical utility

Our hospital has a large enough patient volume (with more than 15,000 births a year) to sponsor and support the present study. We also have the necessary equipment and technical support required for the successful completion of this project. Our research group has conducted a preliminary study on VTE during pregnancy and puerperal periods using a small number of patients. We have thereby begun to understand the occurrence of and risk factors for pregnancy-associated VTE in our country, and possess a solid foundation to pursue future studies where we will revise and evaluate the risk-assessment tool from the RCOG.

## Discussion

Pregnancy and puerperal VTE constitute one of the major causes of maternal mortality. The incidence of VTE during pregnancy has been reported to be 0.5–2.2%,[17] which accounts for 24% of sudden maternal deaths. In pregnancy-associated VTE events, 75–80% have been documented to be DVT and 20–25% to have PE secondary to DVT [1]. 15 % of PE events during pregnancy have been fatal and, among these, 20–30% have resulted in sudden death, [6] with 66% dying within 30 min of the embolism. Studies on pregnancy and puerperal VTE have been performed previously in the United Kingdom, and the RCOG published “Thromboprophylaxis during Pregnancy, Labour and after Vaginal Delivery” in 2004, providing a systematic analysis and evaluation of the risk factors for VTE during pregnancy. Based on these risk factors, appropriate treatments with low-molecular-weight heparin have been administered for thrombosis prophylaxis, which has significantly decreased the maternal death rate caused by VTE. In 2015, ROCG published the third version of “Reducing the Risk of Venous Thromboembolism during Pregnancy and the Puerperium” [13]. Based on a large number of observational studies with reference to data from non-pregnant women, RCOG proposed a VTE risk-assessment-scoring tool during pregnancy and the puerperal periods, with different scores corresponding to different degrees of risk. Women in different pregnancy and puerperal periods should therefore receive corresponding prophylaxis and treatment in order to decrease the incidence of VTE. The United States, Canada, and other countries also conducted a large number studies on pregnancy-associated VTE. ACOG, the American College of Chest Physicians (ACCP), and the Society of Obstetricians & Gynecologists of Canada (SOGC) have developed guidelines for pregnancy-associated VTE, [4, 18, 19] recommending prenatal and puerperal low-molecular-weight heparin prophylaxis for high-risk patients. ACOG specifically recommended that a mechanical pneumatic pump be used at both lower extremities during cesarean section to decrease the incidence of puerperal VTE. Asian countries have also conducted studies on pregnancy-associated VTE. Genetic risk factors—such as antithrombin deficiency, factor V Leiden mutation, and the prothrombin gene G20210A mutation—in Asian populations were significantly lower than in western countries [15]; however, the incidence of VTE is basically the same as in developed countries. In 2016, the International GTH released an expert consensus on the diagnosis and treatment of pregnancy-associated VTE that summarized a large body of evidence proposing a series of protocols to diagnose and treat VTE during pregnancy [8, 20]. In more developed countries, pregnancy-associated VTE is the leading cause of death for pregnant women, while in China, a less-developed country, the leading cause of death during pregnancy is still puerperal hemorrhage. Pregnancy-associated VTE has therefore not received widespread attention from Chinese medical institutions and healthcare providers, although the Chinese Medical Association and Surgical Association have published the third edition of the guidelines for the diagnosis and treatment of DVT. The Chinese Academy of Obstetrics and Gynecology released an expert consensus on the development of DVT and prevention of PE after gynecologic operations in 2017; however, there was no risk assessment or prevention guidelines on pregnancy-associated VTE. Due to the physiologic changes during pregnancy and puerperal periods, guidelines on non-pregnancy-related VTE cannot be directly applied to pregnant women. Moreover, it is not known whether the VTE risk-assessment tool developed in western countries is suitable for the Chinese population. Low-molecular-weight heparin is relatively safe as a preventive medication for maternal DVT, but whether it is appropriate for use in high-risk Chinese patients is also unclear. Therefore, it is imperative that studies based on the Chinese population establish a VTE risk-assessment tool that can provide evidence-based guidelines to prevent and treat pregnancy-associated VTE.

The strengths and limitations of our proposed study are as follows. The first strength is that this is the first cohort study focusing on pregnancy-associated VTE in the Chinese population in Beijing. A second strength is in the innovation in this study in terms of its understanding of the prevalence of pregnancy-associated VTE in China, and the development of a pregnancy-associated VTE risk-assessment tool suitable for the Chinese population. This tool will provide evidence-based guidelines to prevent, diagnose, and treat pregnancy-associated VTE. One limitation of our proposed study is that China has a vast territory, and this study is a single-centered study where the results may not represent the entire Chinese population. Therefore, it will be necessary to conduct a multi-center research study with a larger sample size in the future.

## Data Availability

Data sharing is not applicable to this article as no datasets were generated or analyzed during the current proposal.

## Abbreviations

ACCP: American College of Chest Physicians
ACOG: American College of Obstetricians and Gynecologists
ART: Assisted reproductive technology
CTPA: Computed tomography pulmonary angiography
DVT: Deep vein thrombosis
GTH: Working Group in Women’s Health of the Society of Thrombosis and Haemostasis
IVF: In vitro fertilization
OHSS: Ovarian hyperstimulation syndrome
PE: Pulmonary embolism
RCOG: Royal College of Obstetricians and Gynaecologists
SOGC: Society of Obstetricians & Gynecologists of Canada
V/Q: Ventilation/perfusion
VTE: Venous thromboembolism
